# Editorial: The role of vitamin D in metabolic and cardiovascular health

**DOI:** 10.3389/fnut.2023.1193758

**Published:** 2023-04-24

**Authors:** Ivana Šarac, Marija Djekić-Ivanković, Jasmina Debeljak-Martačić

**Affiliations:** ^1^Institute for Medical Research, National Institute of Republic of Serbia, Centre of Research Excellence in Nutrition and Metabolism, University of Belgrade, Belgrade, Serbia; ^2^School of Population and Global Health, McGill University, Montreal, QC, Canada

**Keywords:** vitamin D, obesity, diabetes, metabolic syndrome, dyslipidemia, NAFLD, cardiovascular disease, cardiometabolic health

In the last few decades, interest in vitamin D (VitD) has grown significantly since numerous studies have suggested that besides its well-established roles in bone metabolism (1), it could have other important roles in organism, including roles in immunity, endocrine, cardiovascular, and reproductive system (2–7). Many studies indicated that VitD status is inversely associated with the incidence of several metabolic diseases and conditions, including obesity (8, 9), insulin resistance (10–14), metabolic syndrome (15–17), dyslipidemia (13, 18, 19), diabetes (10, 11, 20–22), non-alcoholic fatty liver disease (NAFLD) (23–25), and cardiovascular diseases (7, 26–28). However, the findings were often inconsistent, and the cause/effect relationships particularly remained to be confirmed, as well as the molecular pathways of these associations. Moreover, the relationship of VitD with metabolic and cardiovascular disorders seems to be bidirectional: e.g., obesity could worsen VitD deficiency (8), and *vice versa*, VitD deficiency could aggravate obesity and related metabolic and cardiovascular complications (insulin resistance, defects in insulin secretion, disordered metabolism of lipids, hepatic steatosis, and gut dysbiosis) (7, 9) by multiple mechanisms, many of which are still undiscovered and unclarified. Additionally, since in the above-mentioned disorders and diseases there is also chronic inflammation and increased oxidative stress, the role of VitD as immunomodulator and anti-oxidative agent has been proposed as one of the mechanisms by which VitD can influence these conditions (29–34).

This Frontiers Research Topic “*The role of vitamin D in metabolic and cardiovascular health*” focused on epidemiological research on associations of VitD with metabolic and cardiovascular health, particularly in specific population groups, and pathophysiological pathways of these associations, as well as possible confounding factors which modulate these associations. The Research Topic welcomed also articles on the role of VitD as one of supporting therapeutics in cardiovascular and metabolic diseases, as well as immunomodulator.

In this Research Topic there are 13 papers covering the above-mentioned aspects.

Chen Y. C. et al. compared the risk for VitD deficiency across different categories of metabolically healthy normal weight (MHNW) to metabolically unhealthy overweight/obese insulin resistant (MUO) subjects, by studding 6,655 Chinese adults. The study confirmed the highest risk for VitD deficiency among the MUO subjects, but also indicated some gender and age- related differences: among men, the increased risk was noted particularly in MUO men >50 years old, while in younger men, the risk was highest among metabolically healthy obese (MHO) men. In contrast, among women, in both age subgroups the highest risk was represented among MUO women, but the stronger association was noted among younger women. This study indicated possible gender-influenced associations of VitD status with obesity and adverse cardiometabolic and inflammatory profiles.

Similarly, Yin X. et al. analyzed the associations of VitD status with HOMA-IR (Homeostatic Model Assessment of Insulin Resistance), as a robust measure of insulin resistance, in 6,079 American adults without diabetes and other chronic diseases, by using the data from the National Health and Nutrition Examination Surveys (NHANES). The study also confirmed the negative associations between serum VitD concentrations and HOMA-IR, which remained significant after multiple adjustments for many possible confounders, including age, gender, race/ethnicity, and body mass index (BMI). Nevertheless, the further stratification analyses showed some racial/ethical differences: in people with Non-Hispanic Black origin this inverse association between VitD and HOMA-IR was not observed, which indicates the need for further studies focused on ethnic/racial disparities.

Song et al. examined the possible additive effects of obesity and VitD status on the all-cause, cardiovascular and cancer-related mortality, by using the data from the NHANES surveys. In the models adjusted for multiple confounders (including age, gender, race/ethnicity, smoking, and BMI), an independent effect of VitD both insufficiency and deficiency on all mortality rates was confirmed, with deficiency having stronger effect. Interestingly, the effect of VitD deficiency overcame the effect of obesity on all mortality rates. The highest risk for overall and cardiovascular mortality was observed among VitD deficient obese subjects, while for cancer mortality among VitD deficient normal weight subjects, indicating different mechanisms of associations of VitD with mortality in different conditions.

Similarly, Chen X. et al. examined the possible effects of VitD status on the all-cause, cardiovascular and cancer-related mortality among subjects with hyperlipidemia, by using the data from the NHANES surveys. In the models adjusted for multiple confounders (including age, gender, race/ethnicity, smoking, and BMI), serum VitD level was identified as an independent factor for all-cause and cardiovascular mortality, but no association was found with malignancy-specific mortality among these subjects. Particularly serum VitD levels <25 ng/ml were associated with a higher risk for all-cause and cardiovascular mortality, indicating the need for monitoring of VitD levels and correcting VitD insufficiency/deficiency among hyperlipidemic subjects.

Zheng et al. using the NHANES data found a significant negative correlation between serum VitD levels and the risk of frailty in older people.

Shree et al. performed a meta-analysis on the association of serum VitD levels and polymorphism in the VitD receptor (VDR) gene with celiac disease, showing that reduced serum level of 25(OH)D and rs2228570-T polymorphism of *Fok1* T-allele of VDR gene could be implicated in pathophysiology of this autoimmune disease. Zhou et al. examined the association between serum VitD levels and plasma myeloperoxidase (MPO) levels, as a marker of oxidative stress, in 6,414 Chinese women and men. After adjusting for multiple confounders, the study found that circulating 25(OH)D was negatively associated with MPO levels.

Yin W. J. et al. examined 3,713 pregnant Chinese women in the second trimester of pregnancy, their serum VitD levels, biochemical and clinical indicators of cardiovascular risk and inflammation, and the inflammatory potential of their diet, using the empirical dietary inflammatory pattern (EDIP) score. The study revealed that serum VitD levels mediated significant proportion of the association between the dietary inflammatory potential (i.e., EDIP score) and cardiovascular risk in pregnant women. At the same time, the circulating marker of inflammation, high-sensitivity C-reactive protein (hs-CRP), mediated significant proportion of the association between serum VitD levels and cardiovascular risk, indicating significance of anti-inflammatory effect of VitD in the prevention of cardiometabolic disturbances related to pro-inflammatory diets.

Yang et al. performed a meta-analysis on the effects of VitD supplementation on the circulating lipid levels in subjects with prediabetes, and found that VitD supplementation might beneficially affect triglyceride levels in these subjects, while no significant effects on total cholesterol, HDL-cholesterol and LDL-cholesterol were found. The study revealed that particularly longer duration of treatment (more than 1 year), with doses which correct VitD deficiency/insufficiency, are required to improve triglyceride levels. However, just a few studies were included, and more research on that topic is necessary.

An interesting article by Lee et al. focused on the effect of VitD supplementation on hypercalciuria/urolithiasis prevention in 140 children with epilepsy undergoing ketogenic dietary therapy (KDT). It is known that ketogenic diets relate to increased risk for hypercalciuria/urolithiasis, while the role of VitD on this risk is less clear. Interestingly, the study showed an inverse association of serum VitD levels with the urinary calcium/urinary creatinine ratio, a marker of hypercalciuria. The study also pointed-out that the serum VitD levels >40 ng/mL and the vitamin D3 supplementation doses >50 IU/kg are probably needed for preventing hypercalciuria related to KDT.

Bernardo et al. studied the combined effect of obesity (induced by a high-fat diet) and VitD dietary depletion on metabolic profile and progression of kidney damage in an experimental model of ischemia/reperfusion kidney injury in rats. The study pointed out both independent and additive effects of obesity and VitD depletion on exacerbation of multiple metabolic and inflammatory changes, and progression of functional, hemodynamic, and morphological kidney alterations.

Gázquez et al. studied the effect of VitD supplementation during pregnancy in rats by using different VitD metabolites. The study showed that the monohydroxylated form of VitD, 25(OH)D3, given orally provided better VitD availability compared to vitamin D3: it doubled 25(OH)D3 concentrations in maternal and fetal blood. No adverse effects on pregnancy and fetus were shown. Moreover, 25(OH)D3 had an additional effect on the expression of VDR, fatty acid translocase (FAT), and scavenger-receptor class B type-1 (SR-B1) in maternal liver; and VDR and glutamate decarboxylase GAD67 in fetal brain, which requires further investigation.

Finally, Hanel et al. compared the gene-regulatory potential of three different VitD metabolites: 25(OH)D3, 25(OH)D2, and 1,25(OH)2D3 in human peripheral blood mononuclear cells (PBMCs), and found that although monohydroxylated metabolites can have similar effect on expression of 206 common target genes, their effective concentrations were in the range of supra-physiological concentrations and were 600-fold higher than effective concentrations for 1,25(OH)2D3, indicating 600-fold lower effectiveness.

In summary, the articles in this Research Topic confirm the independent and additive role of VitD in pathophysiology of cardiometabolic and autoimmune diseases, and emphasizes the need for further research in this field. Particularly studies in different population groups, studies on pathophysiological mechanisms, and well-controlled randomized trials with VitD as preventive and therapeutic agent, are needed.

## Author contributions

IŠ wrote the manuscript. MD-I and JD-M revised, co-wrote, and edited the manuscript. All authors listed have made a substantial, direct, and intellectual contribution to the work and approved it for publication.
